# Clinical variability in complementary feeding counseling in Türkiye: results from a pediatrician survey

**DOI:** 10.3389/fped.2025.1646667

**Published:** 2025-11-11

**Authors:** Nalan Karabayır, Demet Deniz Bilgin, Mine Başıbüyük, Övgü Büke

**Affiliations:** 1 Social Pediatrics, Health Science Institute, Istanbul Medipol University, Istanbul, Türkiye; 2 Pediatrics Department, International School of Medicine, Istanbul Medipol University, Istanbul, Türkiye; 3 Department of Pediatrics, Istanbul Haseki Training and Research Hospital, University of Health Sciences, Istanbul, Türkiye; 4 Pediatrics Department, Faculty of Medicine, Istanbul Medipol University, Istanbul, Türkiye; 5 Pediatrics Department, Cerrahpaşa Medical Faculty, Istanbul University-Cerrahpaşa, İstanbul, Türkiye

**Keywords:** infant, complementary feeding, food allergies, pediatricians, physicians’ practice patterns

## Abstract

**Introduction:**

Complementary feeding (CF) is a critical period in infant nutrition, during which pediatricians play a pivotal role. This study aimed to examine CF recommendations by pediatricians in Türkiye, focusing on differences based on infants’ allergy status, and the influence of physician characteristics.

**Methods:**

This cross-sectional study was conducted between September and December 2024 using a semi-structured, self-administered online questionnaire developed by the researchers and distributed among pediatric physicians in Türkiye, including residents, general pediatricians, and subspecialists. For the purposes of this study, allergic infants were defined as those with any form of physician-diagnosed allergy (e.g., atopic dermatitis), excluding confirmed specific food allergies.

**Results:**

Among 300 pediatric physicians, 90.3% recommended exclusive breastfeeding for the first six months. For non-allergic infants, 87.7% advised initiating CF at six months, compared to 75% for allergic infants. The most commonly recommended initiation method was spoon-fed purées (54.7%), with vegetables being the most frequently suggested first food (61.3%), followed by yogurt (24.3%) and fruit purée (9.3%). Significant differences were observed between allergic and non-allergic infants in the timing of allergenic food introduction. A ≥3-day interval between new foods was more frequently recommended for allergic infants (91.3% vs. 78.3%, *p* < 0.001). Egg white, egg yolk, and fish were introduced later in allergic infants (*p* < 0.001), while recommendations for cow's milk and cereals did not differ significantly. Baby-led weaning (BLW) and Baby-Led Introduction to Solids (BLISS) were recommended by 76.7% and 38% of respondents, respectively. Female and less experienced physicians were more likely to endorse infant-led feeding approaches like BLW and BLISS, while more experienced physicians preferred spoon-feeding. Multivariate logistic regression revealed that physician experience and gender were significantly associated with CF method recommendations.

**Discussion:**

These findings indicate that although most pediatricians in Türkiye align with international CF guidelines, notable inconsistencies remain based on infant allergy status and provider characteristics, underscoring the need for standardized and evidence-based training programs.

## 1 Introduction

Complementary feeding (CF), defined as the introduction of nutritionally adequate solid and semi-solid foods alongside continued breastfeeding and/or formula feeding, represents a critical developmental milestone that influences infant growth, neurodevelopment, and long-term health outcomes (1, 2). Current international bodies endorse timely CF, yet differ subtly in the recommended age of initiation: The World Health Organization (WHO) advocates exclusive breastfeeding (EBF) for the first six months, followed by CF (3), while the American Academy of Pediatrics (AAP) also supports CF initiation around six months (4). In contrast, the European Society for Paediatric Gastroenterology, Hepatology and Nutrition (ESPGHAN) recommends introducing CF between 4 and 6 months, provided that developmental readiness is observed (5).

Translating CF guidelines into day-to-day practice is entrusted primarily to pediatricians, who remain parents' principal source of evidence-based advice (6, 7). Nevertheless, international surveys consistently demonstrate heterogeneity in clinical recommendations, encompassing both the timing and qualitative composition of first foods (8–10). These variations may arise from cultural factors, clinical experience, personal beliefs, and differing interpretations of the evolving literature (9). Such variability is clinically relevant since inconsistent guidance may undermine parental confidence, contribute to suboptimal micronutrient intake, and delay the development of appropriate feeding skills.

A particularly debated issue in CF is the timing of allergenic food introduction. While it was previously believed that early exposure might increase allergy risk, accumulating evidence now supports early introduction to promote oral tolerance and reduce the likelihood of food allergies (11). Landmark studies have shown that early introduction of peanut and egg can reduce the risk of allergy (12–14). Accordingly, many guidelines recommend introducing these allergens early in life. The European Academy of Allergy and Clinical Immunology (EAACI) advises including well-cooked egg and peanut (in high-risk populations) during complementary feeding (15). The National Institute of Allergy and Infectious Diseases (NIAID) proposes a risk-based approach: for high-risk infants (e.g., severe eczema or egg allergy), peanut should be introduced between 4 and 6 months following allergy testing or specialist consultation; for moderate-risk infants, home introduction around 6 months is acceptable; and for low-risk infants, peanut may be freely introduced alongside other solids (16). In contrast, the 2021 American Academy of Allergy, Asthma, and Immunology; American College of Allergy, Asthma, and Immunology; and the Canadian Society for Allergy and Clinical Immunology (AAAAI/ACAAI/CSACI) consensus advises introducing both peanut and egg between 4 and 6 months for all infants, regardless of risk level, and states that routine screening is not required, though it may be offered based on family preference (17). While specific benefits of early introduction remain unclear for other allergenic foods such as fish or wheat, delaying their introduction offers no advantage and may even increase allergy risk (17–19). Therefore, most expert recommendations emphasize non-delayed introduction of allergenic foods alongside the initiation of CF (5, 17). Nonetheless, delayed introduction is still commonly advised in clinical practice, especially for infants perceived to be at higher risk (8, 10). This discordance highlights an ongoing gap between current scientific evidence and everyday practice, underscoring the need to further explore physicians' attitudes in allergy-related contexts.

Beyond the timing of CF and allergenic food introduction, the method by which CF is implemented also varies. The most common method is traditional spoon-feeding (TSF), which involves starting with purées and gradually advancing to more textured foods (3, 20). Alternatively, baby-led weaning (BLW) promotes infant self-feeding of age-appropriate family foods from around six months and has been associated with improved appetite regulation, greater enjoyment of meals, and, in some studies, with a reduced risk of overweight (21–25). However, concerns have been raised about possible inadequate intake of iron and energy (24). To address these limitations, the BLISS (Baby-Led Introduction to Solids) method was developed. This modified version of the BLW approach emphasizes offering iron-rich, energy-dense, and safe foods. Randomized controlled trials suggest that BLISS supports adequate micronutrient intake, similar BMI trajectories, and no increased risk of choking compared to TSF (26–29).

In Türkiye, national guidelines provide consistent recommendations on infant and young child feeding. Publications from the Ministry of Health including the *Breastfeeding Counseling Implementation Manual*, the *Nutrition Guide for Türkiye*, and the *Follow-up Protocols for Infants, Children, and Adolescents* discourage the introduction of solid foods before six months and emphasize EBF during this period (30–32). CF is recommended to begin at six months with safe, age-appropriate, and nutrient-dense foods, following WHO principles (30–32). Similarly, the *Infant Nutrition Guideline* published by the Turkish Society of Pediatric Gastroenterology, Hepatology and Nutrition recommends introducing CF around six months of age. This guideline states that CF should not begin before four months and should not be delayed beyond six-and-a-half months, aligning closely with ESPGHAN recommendations (33). Although national guidelines in Türkiye do not provide separate recommendations for allergic infants, they offer general guidance on the introduction of allergenic foods. According to *Nutrition Guide for Türkiye*, items such as egg, fish, and gluten-containing foods may be introduced between 6 and 12 months (30). *The Breastfeeding Counseling Implementation Manual* recommends starting allergenic foods from the sixth month, noting that early introduction does not increase allergy risk and may even be protective (32). *The Infant Nutrition Guideline* similarly warns that delaying allergenic foods beyond one year may increase allergy risk, particularly in high-risk infants (33).

Türkiye offers a distinct sociocultural context for examining CF practices. Breastfeeding rates in Türkiye are notably high. According to the 2018 Turkey Demographic and Health Survey (TDHS), 98% of infants were breastfed at some point. The EBF rate in the first month was 59%, and 40.7% in the first six months (34). Compared to global benchmarks in the 2024 Global Breastfeeding Scorecard, Türkiye performs above average in initiating and maintaining breastfeeding, placing it among 23 countries that have increased their EBF rates by more than 10 percentage points since 2017 and already exceeding the 50% EBF target set for 2025 (35). The 2018 TDHS data also show that in Türkiye, 40.5% of breastfed infants at 4–5 months were receiving solid or semi-solid foods, 35.6% consumed dairy products such as yogurt or cheese, and 19.3% ate fruits or vegetables (34). In terms of liquids, 34.4% received infant formula, 13.9% consumed animal milk, and 28.8% were given other non-water liquids (34). Several recent studies support these findings. Kocagözoğlu et al. reported that 38.8% of infants were introduced to CF before six months, while Sezer et al. found a similar rate of 30.6% (36, 37). Such cultural practices, especially the early introduction of yogurt and various liquids, may shape pediatricians' CF recommendations.

Despite the growing body of literature on CF, to date, no nationwide study has systematically investigated whether pediatricians in Türkiye differentiate their CF recommendations based on infants' allergy status. Moreover, little is known about how physician-level factors, such as gender, years of experience, practice setting, or professional role (resident, general pediatrician, or subspecialist) influence their CF recommendations. In response to this gap, the present cross-sectional study was conducted to: (i) characterize current CF recommendations among practicing pediatricians in Türkiye; (ii) assess whether these recommendations differ according to infants' allergy status; and (iii) identify physician characteristics associated with divergent feeding practices.

## 2 Materials and methods

### 2.1 Study design, setting and participants

This descriptive, cross-sectional study was conducted between September and December 2024. A semi-structured questionnaire developed by the researchers was administered online via Google Forms. The inclusion criteria were being a practicing pediatric physician (general pediatrician, pediatric subspecialist, or pediatric resident) and currently working in Türkiye. The exclusion criterion was not being actively engaged in clinical practice. In total, eight questionnaires were excluded on this basis, as respondents were retired and not currently practicing, resulting in 300 valid responses included in the analysis. The demographic and professional characteristics of the participants (gender, years of experience, institution type, and title) are presented in Table 1.

**Table 1:** Demographic characteristics of the participants.

Characteristic	*n* (%)	Median (Range)
Age		39 (26–70)
Gender		
Male	93 (31)	
Female	207 (69)	
Title		
Pediatric Resident	36 (12)	
General Pediatrician	229 (76.3)	
Pediatric Subspecialist	35 (11.7)	
Years of Experience		12 (1–46)
<10	101 (33.7)	5 (1–9)
≥10–<20	112 (37.3)	13 (10–19)
≥20	87 (29.0)	27 (20–46)
Institution		
Public	193 (64.3)	
Private	107 (35.7)	

### 2.2 Outcomes, variables and definitions

The primary outcomes were pediatricians’ recommendations regarding the timing of CF, the interval between introducing new foods, and the age of introducing selected foods (egg yolk, egg white, fish, cow's milk, and cereals), assessed separately for allergic and non-allergic infants. In this study, “allergic infants” were defined operationally as those with any type of physician-diagnosed allergic condition (e.g., atopic dermatitis), excluding confirmed specific food allergies, as specified in the questionnaire. This classification was based on the responding physician's clinical judgment, and no formal diagnostic confirmation (e.g., via standardized criteria) was required. Independent variables included the infant's allergic status (allergic vs. non-allergic) and physician characteristics (gender, years of experience, academic title, and type of healthcare institution). Secondary outcomes included CF initiation methods (spoon-feeding, BLW, and BLISS), timing of texture introduction (lumpy foods, finger foods, family meals), preferred initial food groups (e.g., vegetables, fruits, yogurt), and the timing of other complementary foods. Some of these secondary variables were reported descriptively to illustrate general trends across the sample and were not analyzed comparatively by physician characteristics. In contrast, comparative analyses were conducted for breastfeeding recommendations, CF initiation timing and methods, interval between new food introductions, and the recommendation of BLW and BLISS, in relation to physician characteristics.

### 2.3 Data sources and measurement

Data were collected using a self-administered questionnaire developed by the researcher based on current literature and guidelines on CF. To assess clarity and feasibility, a pilot study was conducted with five pediatricians and revisions were made based on their feedback. The final questionnaire included 68 items covering demographics, CF recommendations, preferred initial foods, timing of food introduction, and feeding methods (spoon-feeding, BLW, and BLISS). The questionnaire required approximately 10–15 min to complete.

Recommendations on CF timing and the age of introducing specific foods were measured using predefined multiple-choice categories reflecting age ranges in months. Similarly, the intervals between introducing new foods were assessed using predefined day-range options. Identical question formats and response categories were used for both allergic and non-allergic infant categories to ensure comparability. The timing of introducing different food textures (e.g., lumpy foods, finger foods, family meals) was also captured using categorical age ranges. Feeding method preferences (e.g., spoon-fed purée, BLW, BLISS) were recorded through standardized categorical responses.

### 2.4 Sample size and sampling method

The required sample size was calculated using G*Power (version 3.1.9.7) for a chi-square goodness-of-fit test, used as a proxy for the McNemar–Bowker test of symmetry, which is not directly supported by the software. Based on a medium effect size (*w* = 0.3), *α* = 0.05, power = 0.80, and 10 degrees of freedom (corresponding to a 5 × 5 contingency table), the minimum sample size was determined to be 181 participants. Participants were recruited through invitations shared in pediatricians' WhatsApp groups. A snowball sampling method was employed, in which participants were invited to share the survey within their professional networks. Participation was voluntary, and informed consent was obtained prior to participation. Responses were anonymized to ensure confidentiality and minimize potential response bias.

### 2.5 Statistical analysis

Data were analyzed using IBM SPSS Statistics for macOS, version 30.0 (IBM Corp., Armonk, NY, USA). Descriptive statistics were reported as means ± standard deviations (SD) or medians (min–max) for continuous variables, and as frequencies and percentages for categorical variables. Within-subject comparisons of pediatricians' recommendations for allergic and non-allergic infants were conducted using the McNemar–Bowker test, which is appropriate for detecting shifts across matched conditions when response options involve more than two categories. Associations between physician characteristics and CF recommendations were examined using chi-square or Fisher's exact test, depending on cell frequencies. A *p*-value of <0.05 was considered statistically significant. Additionally, multivariate logistic regression analyses were performed with gender, academic title, years of experience, and institution type as independent variables. Results were presented as odds ratios (ORs) with 95% confidence intervals.

### 2.6 Ethical approval

This study was approved by the Ethics Committee of Istanbul Medipol University (*E-10840098-202.3.02-5328*).

## 3 Results

### 3.1 Demographic features

A total of 300 pediatricians were included in the analysis. The participants' median age was 39 years (range: 26–70 years), and 69% were female. Most were general pediatricians (76.3%), followed by pediatric residents (12%), and pediatric subspecialists (11.7%). Regarding professional experience, 33.7% had less than 10 years, 37.3% had between 10 and 20 years, and 29.0% had 20 years or more of experience. Of the participants, 64.3% worked in public institutions and 35.7% in private settings. Detailed demographic characteristics are presented in Table 1.

### 3.2 Recommendations on breastfeeding duration and the timing of complementary feeding

The majority of pediatricians (90.3%) recommended EBF for the first six months, and 77.3% recommended continuing up to 24 months. For non-allergic infants, 87.7% recommended initiating CF at six months of age. Additionally, a majority (78.3%) advised introducing new foods at intervals of at least three days. For allergic infants, 75% recommended starting CF at six months, and 91.3% advised a minimum interval of three days between new food introductions. Details of pediatricians' recommendations on exclusive and total breastfeeding durations, as well as the timing of CF initiation, are summarized in Table 2.

**Table 2:** Pediatricians’ recommendations on breastfeeding and complementary feeding initiation.

Parameter	*n* (%)
Exclusive breastfeeding duration	
4 mo	4 (1.3)
4–6 mo	25 (8.3)
6 mo	271 (90.3)
Total breastfeeding duration	
12–24 mo	18 (6.0)
24 mo	232 (77.3)
At least 24 mo	34 (11.3)
>24 mo	13 (4.3)
Other	3 (1.0)
Timing of CF (non-allergic Infants)	
<4 mo	0 (0.0)
4 mo	5 (1.7)
5 mo	18 (6.0)
6 mo	263 (87.7)
>6 mo	14 (4.6)
Timing of CF (allergic infants)	
<4 mo	1 (0.3)
4 mo	14 (4.7)
5 mo	31 (10.3)
6 mo	225 (75.0)
>6 mo	29 (9.6)
Interval between new foods (non-allergic infants)	
No specific interval recommended	35 (11.7)
1 d	9 (3.0)
2 d	21 (7.0)
≥3 d	235 (78.3)
Interval between new foods (Allergic infants)	
No specific interval recommended	17 (5.7)
1 d	6 (2.0)
2 d	3 (1.0)
≥3 d	274 (91.3)

mo, month; d, day; CF, complementary feeding.

### 3.3 Recommendations for types and introduction timing of complementary foods

Vegetables were the most recommended starting foods (61.3%), followed by yogurt (24.3%) and fruit purée (9.3%). The most preferred initial vegetables were carrot (86.7%), zucchini (83.3%), potato (77.7%), pumpkin (34%) and sweet potato (30.3%). Among fruits, apple (90%), banana (64.7%), pear (56.7%), peach (53.2%), and avocado (24%) were most recommended. Table 3 summarizes recommended initial foods and food groups.

**Table 3:** Recommended foods and food groups in complementary feeding.

Parameter	*n* (%)
Initial food groups recommended for CF	
Vegetables	184 (61.3)
Yogurt	73 (24.3)
Fruit purée	28 (9.3)
Fruit juice	7 (2.3)
Cereals	7 (2.3)
Egg yolk	1 (0.3)
Most recommended vegetables	*n* (%)*
Carrot	260 (86.7)
Zucchini	250 (83.3)
Potato	233 (77.7)
Pumpkin	102 (34.0)
Sweet Potato	91 (30.3)
Most Recommended Fruits	*n* (%)*
Apple	270 (90.0)
Banana	194 (64.7)
Pear	170 (56.7)
Peach	159 (53.2)
Avocado	72 (24)
Recommendation of various foods	*n* (%)
Red meat	298 (99.3)
Fish	291 (97.0)
Cereals	274 (91.3)
Chicken	234 (78.0)
Nuts	190 (63.3)
Foods containing refined sugar	13 (4.3)
Cow's milk	148 (49.3)
Plant-based milk	39 (13.0)
Black tea	39 (13)
Herbal teas	196 (65.3)
Mineral water	142 (47.3)

* Participants could select more than one option; therefore, percentages may exceed 100%.

**Figure 1:**
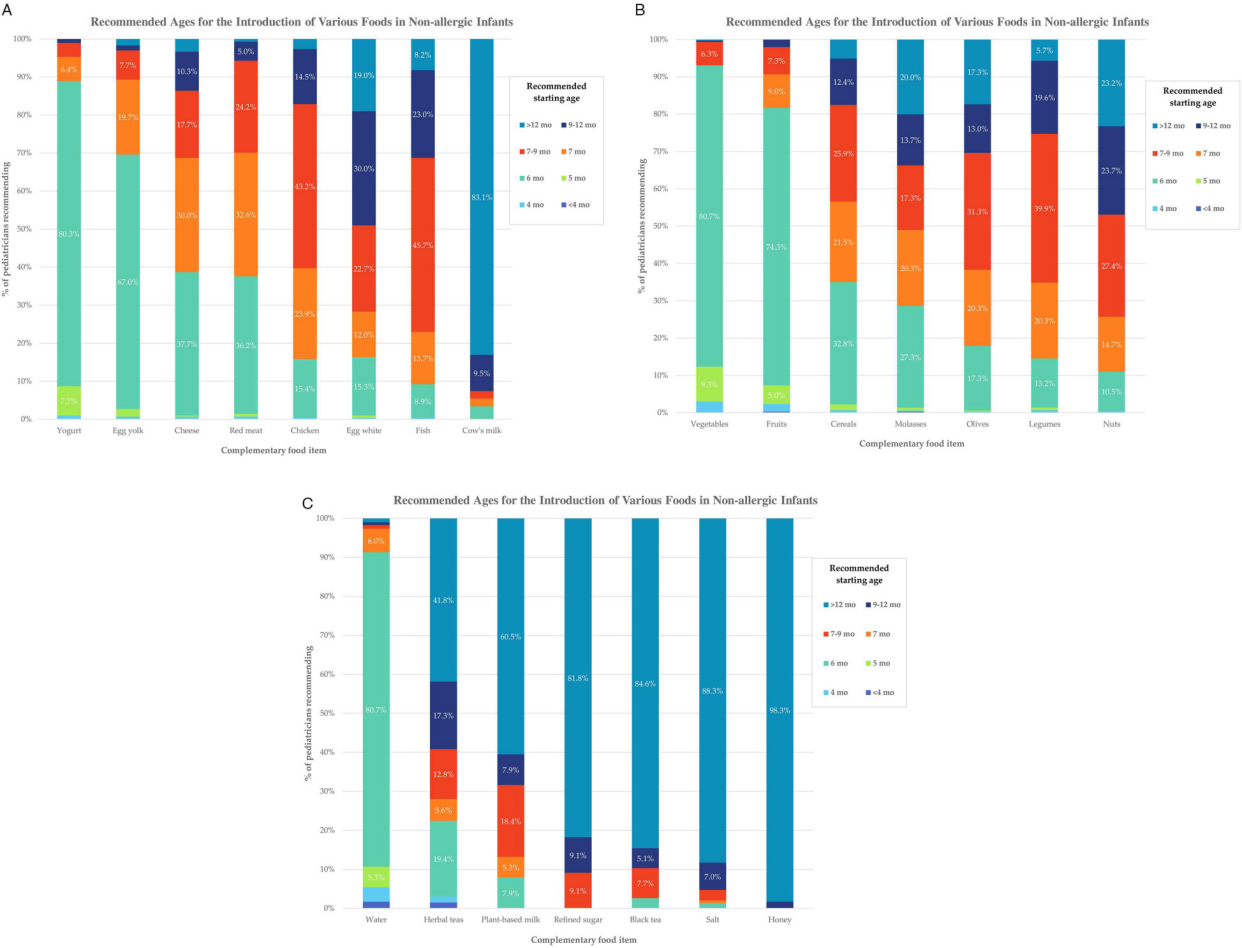
Recommended ages for the introduction of various complementary foods in non-allergic infants, according to pediatricians’ responses. **(A–C)** The figures show the percentage of respondents advising the introduction of each food item across specific age intervals. Each bar represents the distribution of recommendations for that specific food item, numerical percentages are shown for values ≥5%. Detailed data are available in Supplementary Table S1.

The most commonly recommended age for introducing egg yolk was 6 months (67.0%), while egg white was most often suggested between 9 and 12 months (30%). Red meat was recommended by 99.3% of pediatricians, typically at 6 months (36.2%) and 7 months (32.6%). Fish was recommended by 97.0%, with 45.7% advising introduction between 7 and 9 months. Cereals were recommended by 91.3%, mainly at 6 months (32.8%). Cow's milk was recommended by 49.3%, with 83.1% suggesting it after 12 months. Water was generally recommended starting at six months (80.7%). Black tea, a beverage traditionally consumed in Türkiye, was recommended by 13%, predominantly after 12 months. Mineral water was recommended by 47.3%, with the earliest suggested age being six months. Figure 1 illustrates pediatricians' recommended timing for the introduction of various complementary foods in non-allergic infants. A detailed breakdown of response distributions by food item is provided in Supplementary Table S1.

### 3.4 Recommendations on the Introduction of complementary foods in allergic children

**Figure 2:**
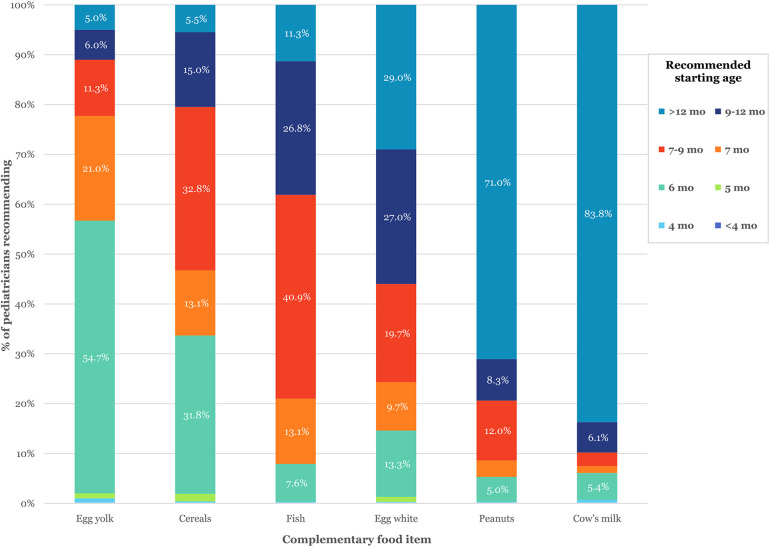
Recommended ages for the introduction of various complementary foods in allergic infants, according to pediatricians’ responses. The figure displays the percentage of respondents advising the introduction of selected foods at specific age intervals. Each bar represents the distribution of recommendations for that specific food item, numerical percentages are shown for values ≥5%. Detailed response breakdowns are provided in Supplementary Table S2.

For allergic children, 54.7% of pediatricians recommended egg yolk at 6 months. Egg white was mostly advised at 9–12 months (27%) or after 12 months (29%). Cow's milk (83.8%) and peanuts (71%) were primarily recommended after 12 months, while cereals (32.3%) and fish (40.9%) were typically introduced at 7–9 months. Recommended starting ages for various foods in allergic infants are illustrated in Figure 2. (A detailed breakdown of response distributions by food item is provided in Supplementary Table S2).

### 3.5 Comparison of recommendations between allergic and Non-allergic children

**Figure 3:**
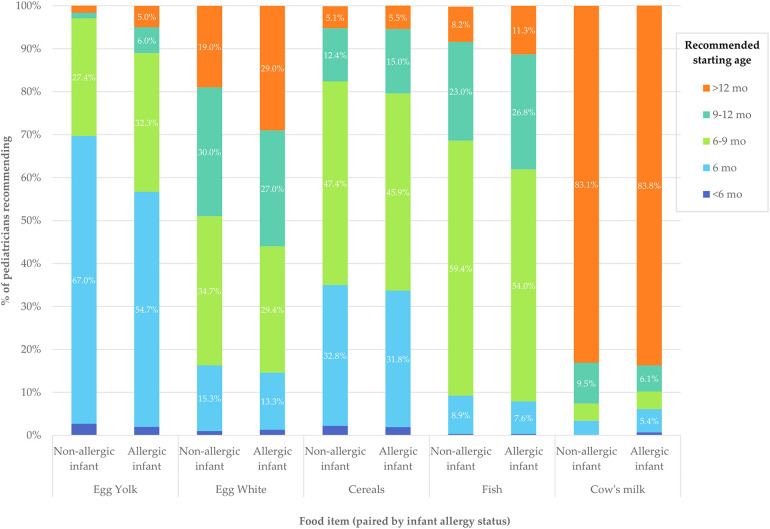
Recommended starting ages for selected foods in allergic and non-allergic infants, according to pediatricians’ responses. The figure compares the percentage of pediatricians recommending the introduction of egg yolk, egg white, cereals, fish, and cow's milk at specific age intervals. For each food item, bars are paired as non-allergic and allergic infant. Each bar represents the distribution of responses for a given food and allergy status, numerical percentages are shown for values ≥5%. Detailed numerical values are available in Supplementary Table S3.

Pediatricians' CF recommendations differed significantly for allergic and non-allergic infants. While most advised starting CF at 6 months for non-allergic children, responses for allergic children varied more, with shifts toward both earlier and later months (*p* < 0.001). A longer interval between introducing new foods (≥3 days) was also more commonly recommended for allergic infants (*p* < 0.001). There was a clear tendency to postpone the introduction of egg yolk and egg white in allergic children compared to non-allergic children (*p* < 0.001). A similar, though more modest shift was observed in fish recommendations (*p* = 0.040). In contrast, recommendations for cow's milk and cereals were similar regardless of allergy status. (*p* = 0.334 and *p* = 0.494 respectively). Table 4 summarizes the statistical significance of differences in CF recommendations by allergy status. Figure 3 illustrates the distribution of recommended food introduction ages across allergic and non-allergic infants. A more detailed breakdown of matched pediatrician responses -including specific shifts across age categories- is available in Supplementary Table S3.

**Table 4:** Comparison of complementary feeding recommendations according to allergy status.

Recommendation	*n*	McNemar– Bowker *χ*^2^	df	*p*-value
Timing of complementary feeding	300	24.5	3	<0.001
Interval between new foods	300	36.372	3	<0.001
Egg Yolk introduction age	300	45.789	8	<0.001
Egg White introduction age	300	32.349	9	<0.001
Fish introduction age	291	13.192	6	0.040
Cow's milk introduction age	148	3.400	3	0.334
Cereal introduction age	274	6.397	7	0.494

### 3.6 Recommendations on methods for complementary feeding

The most frequently recommended approach was starting CF with spoon-fed purees (54.7%), followed by a combination of spoon-feeding and BLW (25.3%). Only 1.0% of respondents recommended initiating CF solely with BLW. Regarding food textures, lumpy foods were most commonly suggested between 6 and 9 months (55.7%). Finger foods were most frequently recommended between 9 and 12 months (55.3%), while 29.0% suggested starting at 6–9 months, and 10.7% after 12 months. For transition to family meals, 45.3% of pediatricians recommended this after 12 months, followed by 31.7% recommending at 9–12 months, and only 13.7% at 6 months.

The BLW method was recommended by 76.7%, and BLISS by 38%. More than half of respondents (55.6%) reported being unfamiliar with the BLISS approach. Although 9–12 months was the most frequently recommended period for initiating both methods, recommendations spanned a wide range, with a substantial proportion advising initiation at 6–9 months. Table 5 summarizes pediatricians' recommendations on complementary feeding methods, including preferred timing.

**Table 5:** Recommendations for complementary feeding methods.

Parameter	*n* (%)
CF Initiation method	
BLW	3 (1.0)
Spoon-fed as lumpy	57 (19.0)
BLW and spoon-feeding	76 (25.3)
Spoon-fed as purée	164 (54.7)
Introduction of lumpy foods	
6 mo	69 (23.0)
6–9 mo	167 (55.7)
9–12 mo	61 (20.3)
>12 mo	3 (1.0)
Introduction of finger foods	
6 mo	15 (5.0)
6–9 mo	87 (29.0)
9–12 mo	166 (55.3)
>12 mo	32 (10.7)
Transition to family meals	
6 mo	41 (13.7)
6–9 mo	28 (9.3)
9–12 mo	95 (31.7)
>12 mo	136 (45.3)
Recommendation of BLW	
Yes	230 (76.7)
No	70 (23.3)
Recommended age for BLW	
6 mo	47 (20.7)
6–9 mo	69 (30.4)
9–12 mo	96 (42.3)
>12 mo	15 (6.6)
Recommendation of BLISS	
Yes	114 (38.0)
No	19 (6.3)
Unfamiliar	167 (55.6)
Recommended age for BLISS	
6 mo	25 (22.3)
6–9 mo	31 (27.7)
9–12 mo	45 (40.2)
>12 mo	11 (9.8)

mo, month; CF, complementary feeding; BLW, baby-led weaning; BLISS, Baby-Led Introduction to SolidS.

### 3.7 Influence of demographic characteristics on complementary feeding recommendations

Physicians' demographic characteristics showed no significant association with breastfeeding duration, timing of CF or intervals for introducing new foods. However, gender, years of experience, and professional title were significantly associated with CF initiation methods and the recommendation of BLW/BLISS approaches (*p* < 0.05). Details are presented in Supplementary Tables S4 and S5.

Multivariate logistic regression models were conducted to examine the influence of physician characteristics on CF recommendations. The model for general recommendation of BLW was statistically significant [*χ*^2^(6) = 28.79, *p* < 0.001; Nagelkerke *R*^2^ = 0.138], as were the models for recommending a combined method involving BLW and spoon-fed purée [*χ*^2^(6) = 19.30, *p* = 0.004; *R*^2^ = 0.091] and for recommending spoon-fed puree only initiation [*χ*^2^(6) = 30.25, *p* < 0.001; *R*^2^ = 0.128]. The model predicting BLISS recommendation demonstrated borderline significance [*χ*^2^(6) = 12.52, *p* = 0.051; *R*^2^ = 0.056]. All models showed acceptable goodness-of-fit according to Hosmer–Lemeshow tests (spoon-fed only: *p* = 0.303; BLW: *p* = 0.317; combined method: *p* = 0.432; BLISS: *p* = 0.955), and their explanatory power ranged from 0.056 to 0.138. Full model statistics are available in Supplementary Table S6.

Regression results indicated that physician experience and gender were significantly associated with CF recommendations. Regarding general endorsement of BLW, physicians with 10–20 years or less than 10 years of experience were significantly more likely to recommend BLW compared to those with ≥20 years (OR = 4.9 and 3.4, respectively; both *p* < 0.001). When asked about preferred initiation methods**,** less experienced physicians were also more likely to support a combined approach involving both BLW and spoon-feeding puree (OR = 4.4 and 2.6; *p* < 0.001 and 0.018, respectively), and less likely to recommend exclusive spoon-fed purées (OR = 0.232 and 0.464; *p* < 0.001 and 0.016). Female physicians were significantly less likely to recommend spoon-feeding only initiation (OR = 0.46, *p* = 0.006), and although not statistically significant, showed a trend toward recommending the combined method (OR = 1.76, *p* = 0.085). Regarding professional title, only the recommendation of the BLISS method was associated with a significant difference: pediatric residents were 5.6 times more likely to recommend BLISS than pediatric subspecialists (95% CI, 1.85–16.96, *p* = 0.002). No significant associations were found between practice setting (public vs. private) and any CF recommendation outcome. Detailed results of multivariate logistic regression analyses are presented in Table 6.

**Table 6:** Logistic regression analysis of factors influencing complementary feeding recommendations.

Predictor	BLW OR (95% CI), *p*	BLISS OR (95% CI), *p*	BLW + Spoon fed puree OR (95% CI), *p*	Spoon-fed puree only OR (95% CI), *p*
Gender (Female vs. Male)	1.68 (0.91–3.10), 0.099	1.25 (0.73–2.16), 0.422	1.76 (0.93–3.35), 0.085	0.46 (0.26–0.80), 0.006
Resident vs. Subspecialist	2.06 (0.56–7.64), 0.278	**5.60 (1.85–16.98), 0.002**	0.56 (0.18–1.80), 0.332	0.56 (0.18–1.80), 0.332
Specialist vs. Subspecialist	1.51 (0.67–3.40), 0.318	1.56 (0.68–3.57), 0.291	0.79 (0.33–1.89), 0.596	0.79 (0.33–1.89), 0.596
10–20 yrs vs. ≥20 yrs	**4.93 (2.12–11.45), <0.001**	0.74 (0.36–1.49), 0.395	**4.36 (1.88–10.12), <0.001**	**0.23 (0.11–0.48), <0.001**
<10 yrs vs. ≥20 yrs	**3.39 (1.70–6.76), <0.001**	0.83 (0.45–1.51), 0.534	**2.55 (1.17–5.55), 0.018**	**0.46 (0.25–0.87), 0.016**
Private vs. Public Institution	1.89 (0.97–3.70), 0.061	1.19 (0.69–2.08), 0.528	1.02 (0.54–1.93), 0.947	1.02 (0.54–1.93), 0.947

Reference groups: Male, subspecialist, ≥20 years of experience, working in public institution.

Bold values indicate statistical significance at *p* < 0.05.

Given the notably high proportion of physicians who reported unfamiliarity with the BLISS method (55.6%), an additional analysis was performed to examine whether this lack of familiarity varied by demographic or professional characteristics. In a binary logistic regression analysis, unfamiliarity with the BLISS method was examined in relation to physician characteristics, including gender, years of experience, institution type, and professional title. The model was not statistically significant overall (Omnibus *χ*^2^ = 4.22, *p* = 0.376; Nagelkerke *R*^2^ = 0.028), and no variables showed statistically significant associations. However, resident physicians showed a borderline lower odds of unfamiliarity compared to subspecialists (OR = 0.36, 95% CI: 0.13–1.02, *p* = 0.054).

## Discussion

To our knowledge, this is the first study to examine CF practices among pediatricians in Türkiye, with a particular focus on variations related to infant allergy status and physician characteristics. Our findings highlight three key themes that reflect both continuity and change in pediatric CF practices in Türkiye. Although the majority of physicians report recommending initiating CF at six months, in line with WHO recommendations, substantial variation persists in the timing of food introduction, especially concerning allergenic foods. Many pediatricians continue to postpone the introduction of eggs and fish in allergic infants, despite accumulating evidence favoring early exposure to reduce allergy risk. Meanwhile, newer trends are emerging among younger and female pediatricians, who appear more open to baby-led feeding methods such as BLW and BLISS. These observations underscore an ongoing tension between evolving scientific evidence and clinical counseling patterns, shaped by individual, generational, and sociocultural dynamics.

Recent literature reveals considerable variability in pediatricians' CF practices regarding the timing. A study among Italian primary care pediatricians found that most recommended starting CF between 4 and 6 months, with a preference for initiation closer to 6 months of age (38). Similarly, other studies reported that recommendations to start CF between the 5th and 6th months outweighed those advising initiation at 5 months (9, 39, 40). In our study, consistent with global recommendations issued by WHO, the majority of participating physicians endorsed EBF for the first six months and recommended initiating CF around six months of age (3). This alignment suggests that international guidelines are increasingly reflected in national pediatric practice.

The WHO advises offering a variety of nutrient-dense complementary foods (41). There is no single “best” first food, as choices depend on cultural norms, food availability, and infant needs (42). Although it is often recommended to start with vegetables, there is no conclusive evidence to support starting with fruits or vegetables first (43). It has been suggested that starting with vegetables reduces babies' preference for sweet tastes and encourages more vegetable consumption later in life, however, these effects may not be long-lasting (8, 44, 45). Our study revealed that vegetables were the most commonly recommended option for initiating CF, followed by yogurt and fruit purée. These findings indicate a strong preference for starting CF with vegetables over fruits but also reflect considerable variation in food selection among physicians. While most pediatricians recommended introducing yogurt as a complementary food around 6 months of age, cow's milk was predominantly advised for use only after 12 months. This distinction is supported by current literature and guidelines, many of which recommend delaying the introduction of unmodified cow's milk as the main drink until after 12 months of age, due to potential adverse effects, particularly iron deficiency anemia (5, 46). In contrast, fermented dairy products such as yogurt, and cheese are generally considered acceptable for introduction between 6 and 12 months of age as part of CF (47). However, some variation exists across guidelines regarding the timing and form of milk-based product introduction (47).

Although current global and national guidelines do not recommend the use of black or herbal teas during infancy, particularly before 12 months of age (3, 33) recent studies in Türkiye have reported that these beverages are commonly consumed. Herbal tea consumption among infants aged 6–24 months has been reported to range between 17% and 30%, while black tea consumption ranges from 10% to 23% (48–51). In our study, although only 13% of pediatricians reported recommending black tea, the majority of these did so after 12 months of age. Herbal teas were recommended more frequently (65.3%), typically between 6 and 12 months. Such findings highlight the need for strategies that support adherence to evidence-based infant feeding guidelines while addressing culturally rooted practices through community-sensitive approaches.

A key finding was the divergence in CF advice provided for allergic vs. non-allergic infants. Current guidelines recommend introducing allergenic foods around six months of age, but not before four months (17). In our study, the introduction of egg yolk, egg white, and fish was later in allergic children, showing a significant difference compared to non-allergic children. However, no significant timing differences were observed for cow's milk or cereals. Similar discrepancies have been reported in Southern European contexts (8, 10). For instance, Capra et al., reported that while egg and fish were generally recommended in the first year of life, their introduction was often delayed in cases with a family history of allergy (10). A study in Greece found that pediatricians recommend longer intervals between introducing new foods in children at high risk of allergy, and tend to delay introduction of allergenic foods such as eggs, seafood and gluten-containing cereals (8). In a U.S. based study involving 563 practitioners, 38.6% recommended waiting at least three days between introducing new foods, whereas 66.3% recommended this interval for infants at risk of developing food allergies (52). Similarly, in our study, a longer interval between introducing new foods was more commonly recommended for allergic infants. Although many physicians advised a minimum three-day gap between new foods, this practice, while pragmatic, is not strongly supported by current evidence (18). There is no standardized definition of an “allergic infant”; guidelines usually describe “high-risk infants” (e.g., severe eczema or egg allergy) or specific diagnoses (15, 16). In our study, the term was operationally defined as any physician-diagnosed allergic condition, other than food allergy. This broad, heterogeneous definition may attenuate between-group differences and make recommendations appear more conservative, which should be taken into account when comparing studies that uses narrower definitions.

While our study did not investigate the specific reasons behind pediatricians' cautious approach to introducing allergenic foods, existing literature highlights several potential barriers. These include parental concerns about allergic reactions, uncertainty among pediatricians regarding guideline implementation, limited consultation time, and inadequate infrastructure for supervised oral food challenges (53, 54). Although awareness of updated guidelines appears high, implementation remains suboptimal in many settings (53). Restricted access to pediatric allergy specialists may also discourage early allergen introduction, particularly in infants perceived to be at higher risk (55). Physicians may adopt defensive practices to mitigate objections, avoid complaints, lengthy trial processes, or other potential threats; however, such defensive medicine practices can carry risks (56). Although direct literature on medicolegal anxiety among pediatricians in this context is limited, it might be one of the contributing factors to the delayed introduction of allergenic foods, even when current guidelines support early introduction in infancy. Importantly, the timing of allergenic food introduction has been shown to be most influenced by physician recommendation (57) underscoring their pivotal role. Yet this cautious approach may inadvertently reduce dietary diversity and contribute to an increased risk of allergy later in childhood.

The multivariate logistic regression analyses highlight how physician-level characteristics influence CF counseling preferences. Pediatricians with fewer than 20 years of experience were significantly more likely to recommend BLW compared to their more experienced colleagues (OR up to 4.9), suggesting a generational shift in favor of infant autonomy. This aligns with literature suggesting generational and gender-based shifts toward infant autonomy, and shared decision-making in pediatric care (10, 58). Similarly, pediatric residents were markedly more likely to recommend the BLISS method compared to pediatric subspecialists (OR = 5.6), likely reflecting their recent training and greater exposure to contemporary feeding paradigms. Interestingly, practice setting (public vs. private) was not associated with any CF outcome, indicating that institutional factors may be less influential than individual clinician beliefs. Although model explanatory power was modest (Nagelkerke *R*^2^ range: 0.056–0.138), key predictors demonstrated consistent directionality, and some models, particularly for BLW and spoon-fed initiation, showed statistical significance. This suggests that provider-level characteristics play a meaningful, though partial, role in shaping infant feeding guidance.

From a health systems perspective, the observed heterogeneity in CF recommendations underscores the need for a national pediatric nutrition framework that not only incorporates the latest scientific evidence but also addresses the sociocultural landscape of Türkiye. National consensus statements, endorsed by relevant professional bodies, could help standardize clinical counseling with international best practices while accounting for local dietary customs and health literacy levels. In addition, integrating CF guidance into pediatric residency curricula and structured continuing medical education programs may enhance the consistency and quality of nutrition-related counseling.

This study has several limitations. As a non-probability approach, snowball sampling introduces potential selection bias, compromises representativeness, and limits generalizability. Although pediatrician-specific data by gender/workplace are publicly unavailable in Türkiye, general physician statistics show 43.2% female (59) and 64.5% in public institutions (60); our sample had 69.0% female and 64.3% in public institutions. While not a definitive benchmark, it provides useful context for representativeness. Professional credentials were not formally verified, which may introduce some uncertainty about sample profile. The questionnaire lacked formal validation, which may have affected clarity and consistency. The broad, operational definition of “allergic infant” introduced heterogeneity that could attenuate observed differences. In addition, reliance on self-reported recommendations rather than verified clinical practice raises the possibility of recall and social desirability biases.

Future research could benefit from moving beyond descriptive cross-sectional designs to explore how CF recommendations are implemented in real-world settings. This includes examining parental adherence and child health outcomes. Moreover, studies assessing the consistency between pediatricians' knowledge and their clinical recommendations, as well as the underlying reasoning, may offer further insight. Qualitative studies into barriers to adopting evidence-based recommendations could help explain the gap between guidelines and clinical implementation and inform strategies to enhance guideline adherence.

## Conclusion

Complementary feeding recommendations of pediatricians in Türkiye are consistent with international guidelines. However, they tend to adopt a more cautious approach for allergic infants, particularly regarding the timing of allergenic food introduction and the interval between new foods. An implication for clinical practice is that, for infants without confirmed food allergy, clinicians should not delay common allergenic foods at CF onset. Notably, a high percentage of pediatricians were unfamiliar with the BLISS method, and less experienced physicians and female physicians were more likely to support baby-led feeding approaches such as BLW and BLISS. These findings highlight the need for standardization of CF recommendations to ensure more consistent practices across providers. Additionally, standardized, evidence-based and culturally sensitive training on CF, particularly addressing allergy-related practices and increasing awareness of infant-led methods, should be promoted to enhance guideline adherence and support informed recommendations in clinical practice.

## Data Availability

The raw data supporting the conclusions of this article will be made available by the authors, without undue reservation.
