# Supplementary material for: Clinical variability in complementary feeding counseling in Türkiye: results from a pediatrician survey
Source: Front Pediatr. 2025 Nov 11;13:1646667. doi: 10.3389/fped.2025.1646667 (PMC12644083; doi:10.3389/fped.2025.1646667)
Supplement: Supplementary file 1 [file Supplementaryfile1.docx]

Supplementary Material

**Table S1.** Recommended Starting Age for Various Complementary Foods

|  | **Recommended Age for Introduction n (%)** | | | | | | | |
| --- | --- | --- | --- | --- | --- | --- | --- | --- |
| **Food** | **<4 mo** | **4 mo** | **5 mo** | **6 mo** | **7 mo** | **7–9 mo** | **9–12 mo** | **>12 mo** |
| Vegetables | 0 (0.0) | 9 (3.0) | 28 (9.3) | 242 (80.7) | 0 (0.0) | 19 (6.3) | 1 (0.3) | 1 (0.3) |
| Fruits | 1 (0.3) | 6 (2.0) | 15 (5.0) | 223 (74.3) | 27 (9.0) | 22 (7.3) | 6 (2.0) | 0 (0.0) |
| Egg yolk | 0 (0.0) | 2 (0.7) | 6 (2.0) | 201 (67.0) | 59 (19.7) | 23 (7.7) | 4 (1.3) | 5 (1.7) |
| Egg white | 0 (0.0) | 1 (0.3) | 2 (0.7) | 46 (15.3) | 36 (12.0) | 68 (22.7) | 90 (30) | 57 (19) |
| Yogurt | 0 (0.0) | 3 (1.0) | 23 (7.7) | 240 (80.3) | 19 (6.4) | 11 (3.7) | 3 (1.0) | 0 (0.0) |
| Red meat | 0 (0.0) | 2 (0.7) | 2 (0.7) | 108 (36.2) | 97 (32.6) | 72 (24.2) | 15 (5.0) | 2 (0.7) |
| Fish | 0 (0.0) | 1 (0.3) | 0 (0.0) | 26 (8.9) | 40 (13.7) | 133 (45.7) | 67 (23) | 24 (8.2) |
| Chicken | 0 (0.0) | 1 (0.4) | 0 (0.0) | 36 (15.4) | 56 (23.9) | 101 (43.2) | 34 (14.5) | 6 (2.6) |
| Cereals | 0 (0.0) | 2 (0.7) | 4 (1.5) | 90 (32.8) | 59 (21.5) | 71 (25.9) | 34 (12.4) | 14 (5.1) |
| Legumes | 0 (0.0) | 2 (0.7) | 2 (0.7) | 39 (13.2) | 60 (20.3) | 118 (39.9) | 58 (19.6) | 17 (5.7) |
| Cheese | 0 (0.0) | 2 (0.7) | 1 (0.3) | 113 (37.7) | 90 (30.0) | 53 (17.7) | 31 (10.3) | 10 (3.3) |
| Olives | 0 (0.0) | 1 (0.3) | 1 (0.3) | 52 (17.3) | 61 (20.3) | 94 (31.3) | 39 (13.0) | 52 (17.3) |
| Molasses | 1 (0.3) | 1 (0.3) | 2 (0.7) | 82 (27.3) | 61 (20.3) | 52 (17.3) | 41 (13.7) | 60 (20.0) |
| Honey | 0 (0.0) | 0 (0.0) | 0 (0.0) | 0 (0.0) | 0 (0.0) | 0 (0.0) | 5 (1.7) | 295 (98.3) |
| Cow's milk | 0 (0.0) | 0 (0.0) | 0 (0.0) | 5 (3.4) | 3 (2.0) | 3 (2.0) | 14 (9.5) | 123 (83.1) |
| Plant-based milk | 0 (0.0) | 0 (0.0) | 0 (0.0) | 3 (7.9) | 2 (5.3) | 7 (18.4) | 3 (7.9) | 23 (60.5) |
| Nuts | 0 (0.0) | 1 (0.5) | 0 (0.0) | 20 (10.5) | 28 (14.7) | 52 (27.4) | 45 (23.7) | 44 (23.2) |
| Salt | 0 (0.0) | 0 (0.0) | 0 (0.0) | 4 (1.3) | 2 (0.7) | 8 (2.7) | 21 (7.0) | 265 (88.3) |
| Refined sugar | 0 (0.0) | 0 (0.0) | 0 (0.0) | 0 (0.0) | 0 (0.0) | 1 (9.1) | 1 (9.1) | 9 (81.8) |
| Water | 5 (1.7) | 11 (3.7) | 16 (5.3) | 242 (80.7) | 18 (6.0) | 3 (1.0) | 2 (0.7) | 3 (1.0) |
| Black tea | 0 (0.0) | 0 (0.0) | 0 (0.0) | 1 (2.6) | 0 (0.0) | 3 (7.7) | 2 (5.1) | 33 (84.6) |
| Herbal teas | 3 (1.5) | 3 (1.5) | 0 (0.0) | 38 (19.4) | 11 (5.6) | 25 (12.8) | 34 (17.3) | 82 (41.8) |

### mo: month; n (%): number and proportion of pediatricians recommending introduction at each age

**Table S2.** Recommended Starting Age for Various Complementary Foods in Allergic Infants

|  | **Recommended Age for Introduction n (%)** | | | | | | | |
| --- | --- | --- | --- | --- | --- | --- | --- | --- |
| **Food** | **<4 mo** | **4 mo** | **5 mo** | **6 mo** | **7 mo** | **7–9 mo** | **9–12 mo** | **>12 mo** |
| Egg yolk | 0 (0.0) | 3 (1.0) | 3 (1.0) | 164 (54.7) | 63 (21.0) | 34 (11.3) | 18 (6.0) | 15 (5.0) |
| Egg white | 0 (0.0) | 1 (0.3) | 3 (1.0) | 40 (13.3) | 29 (9.7) | 59 (19.7) | 81 (27.0) | 87 (29.0) |
| Cow’s milk | 0 (0.0) | 1 (0.7) | 0 (0.0) | 8 (5.4) | 2 (1.4) | 4 (2.7) | 9 (6.1) | 124 (83.8) |
| Fish | 0 (0.0) | 1 (0.3) | 0 (0.0) | 22 (7.6) | 38 (13.1) | 119 (40.9) | 78 (26.8) | 33 (11.3) |
| Cereals | 0 (0.0) | 1 (0.4) | 4 (1.5) | 87 (31.8) | 36 (13.1) | 90 (32.8) | 41 (15.0) | 15 (5.5) |
| Peanuts | 0 (0.0) | 1 (0.3) | 0 (0.0) | 15 (5.0) | 10 (3.3) | 36 (12.0) | 25 (8.3) | 213 (71.0) |

mo: month; n (%): number and proportion of pediatricians recommending introduction at each age

**Table S3.** Matched Transition Matrices Comparing CF Recommendations in Allergic and Non-Allergic Infants

| **Complementary Feeding Start Time** | | | | | | | | | | | | | | |
| --- | --- | --- | --- | --- | --- | --- | --- | --- | --- | --- | --- | --- | --- | --- |
|  | | | |  | **Allergic** | | | | | | | | |  |
|  | | | |  | **<6 mo** | | **6 mo** | | | | **>6 mo** | | | **McNemar–Bowker tests** |
| **Non-allergic** | | | | **<6 mo** | 15 | | 6 | | | | 2 | | | χ² = 24.500  df = 3  p < 0.001 |
|  |  |  |  | **6 mo** | 30 | | 214 | | | | 19 | | |  |
|  |  |  |  | **>6 mo** | 1 | | 5 | | | | 8 | | |  |
| **Interval Between New Foods** | | | | | | | | | | | | | | |
|  | | |  | | **Allergic** | | | | | | | | |  |
|  | | |  | | **<3 d** | | **≥3 d** | | | | **No specific interval** | | | **McNemar–Bowker tests** |
| **Non-allergic** | | | **<3 d** | | 8 | | 21 | | | | 1 | | | χ² = 36.372  df = 3  p < 0.001 |
|  |  |  | **≥3 d** | | 1 | | 233 | | | | 1 | | |  |
|  |  |  | **No specific interval** | | 0 | | 20 | | | | 15 | | |  |
| **Egg Yolk Introduction** | | | | | | | | | | | | | | |
|  |  | | | | **Allergic** | | | | | | | | |  |
|  |  | | | | **<6** | **6** | | | **6–9** | **9–12** | | | **>12** | **McNemar–Bowker tests** |
| **Non-allergic** | **<6** | | | | 4 | 3 | | | 0 | 0 | | | 1 | χ² = 45.789  df = 8  p < 0.001 |
|  | **6** | | | | 2 | 156 | | | 34 | 3 | | | 6 |  |
|  | **6–9** | | | | 0 | 4 | | | 62 | 11 | | | 5 |  |
|  | **9–12** | | | | 0 | 0 | | | 1 | 3 | | | 0 |  |
|  | **>12** | | | | 0 | 1 | | | 0 | 1 | | | 3 |  |
| **Egg White Introduction** | | | | | | | | | | | | | | |
|  |  | | | | **Allergic** | | | | | | | | |  |
|  |  | | | | **<6** | **6** | | | **6–9** | **9–12** | | | **>12** | **McNemar–Bowker tests** |
| **Non-allergic** | **<6** | | | | 1 | 2 | | | 0 | 0 | | | 0 | χ² = 32.349  df = 9  p < 0.001 |
|  | **6** | | | | 1 | 29 | | | 8 | 4 | | | 4 |  |
|  | **6–9** | | | | 1 | 7 | | | 67 | 24 | | | 5 |  |
|  | **9–12** | | | | 1 | 2 | | | 12 | 51 | | | 24 |  |
|  | **>12** | | | | 0 | 0 | | | 1 | 2 | | | 54 |  |
| **Fish Introduction** | | | | | | | | | | | | | | |
|  |  | | | | **Allergic** | | | | | | | | |  |
|  |  | | | | **<6** | **6** | | | **6–9** | **9–12** | | | **>12** | **McNemar–Bowker tests** |
| **Non-Allergic** | **<6** | | | | 1 | 0 | | | 0 | 0 | | | 0 | χ² = 13.192  df = 6  p = 0.040 |
|  | **6** | | | | 0 | 18 | | | 4 | 2 | | | 2 |  |
|  | **6–9** | | | | 0 | 4 | | | 143 | 22 | | | 4 |  |
|  | **9–12** | | | | 0 | 0 | | | 10 | 49 | | | 8 |  |
|  | **>12** | | | | 0 | 0 | | | 0 | 5 | | | 19 |  |
| **Cereal Introduction** | | | | | | | | | | | | | | |
|  |  | | | | **Allergic** | | | | | | | | |  |
|  |  | | | | **<6** | **6** | | | **6–9** | **9–12** | | | **>12** | **McNemar–Bowker tests** |
| **Non-allergic** | **<6** | | | | 4 | 2 | | | 0 | 0 | | | 0 | χ² = 6.397  df = 7  p = 0.494 |
|  | **6** | | | | 0 | 68 | | | 18 | 4 | | | 0 |  |
|  | **6–9** | | | | 1 | 15 | | | 94 | 17 | | | 3 |  |
|  | **9–12** | | | | 0 | 2 | | | 10 | 17 | | | 5 |  |
|  | **>12** | | | | 0 | 0 | | | 4 | 3 | | | 7 |  |
| **Cow's Milk Introduction** | | | | | | | | | | | | | | |
|  | |  | | | **Allergic** | | | | | | | | |  |
|  | |  | | | **<9** | | | **9–12** | | | | **>12** | | **McNemar–Bowker tests** |
| **Non-allergic** | | **<9** | | | 7 | | | 0 | | | | 4 | | χ² = 3.400  df = 3  p = 0.334 |
|  |  | **9–12** | | | 2 | | | 6 | | | | 6 | |  |
|  |  | **>12** | | | 3 | | | 6 | | | | 114 | |  |

**Table S4.** Complementary Feeding Recommendations by Physician Gender and Years of Experience

|  | **Gender n (%)** | |  | **Years of Experience n (%)** | | |  |
| --- | --- | --- | --- | --- | --- | --- | --- |
|  | **Male** | **Female** | ***p* value** | **<10** | **≥10 - <20** | **≥20** | ***p* value** |
| **Exclusive Breastfeeding Duration** |  |  |  |  |  |  |  |
| 4-6 mo | 11 (11.8) | 18 (8.7) | 0.396 | 12 (11.9) | 9 (8.0) | 8 (9.2) | 0.623 |
| 6 mo | 82 (88.2) | 189 (91.3) |  | 89 (88.1) | 103 (92.0) | 79 (90.8) |  |
| **Total Breastfeeding Duration** |  |  |  |  |  |  |  |
| 12-24 mo | 9 (9.7) | 9 (4.3) | 0.054 | 8 (7.9) | 3 (2.7) | 7 (8.0) | 0.084 |
| 24 mo | 76 (81.7) | 156 (75.4) |  | 81 (80.2) | 83 (74.1) | 68 (78.2) |  |
| At least 24 | 6 (6.5) | 28 (13.5) |  | 8 (7.9) | 20 (17.9) | 6 (6.9) |  |
| >24 | 2 (2.2) | 11 (5.3) |  | 3 (3.0) | 5 (4.5) | 5 (5.7) |  |
| Other ^†^ | 0 (0.0) | 3 (1.4) |  | 1 (1.0) | 1 (0.9) | 1 (1.1) |  |
| **Timing of CF (non-allergic)** |  |  |  |  |  |  |  |
| <6 mo | 10 (10.8) | 13 (6.2) | 0.173 | 7 (6.9) | 9 (8.0) | 7 (8.0) | 0.777 |
| 6 mo | 81 (87.1) | 182 (87.9) |  | 91 (90.1) | 98 (87.5) | 74 (85.1) |  |
| >6 mo | 2 (2.2) | 12 (5.8) |  | 3 (3.0) | 5 (4.5) | 6 (6.9) |  |
| **Timing of CF (allergic)** |  |  |  |  |  |  |  |
| <6 mo | 17 (18.3) | 29 (14,1) | 0.233 | 15 (14.9) | 13 (11.6) | 18 (20.7) | 0.171 |
| 6 mo | 64 (68.8) | 161 (77.8) |  | 73 (72.3) | 91 (82.1) | 60 (69.0) |  |
| >6 mo | 12 (12.9) | 17 (8.2) |  | 13 (12.9) | 7 (6.3) | 9 (10.3) |  |
| **Interval Between New Foods**  **(non-allergic)** |  |  |  |  |  |  |  |
| <3 d | 6 (6.5) | 24 (11.6) | 0.262 | 7 (6.9) | 17 (15.2) | 6 (6.9) | 0.124 |
| ≥3 d | 78 (83.9) | 157 (75.8) |  | 84 (83.2) | 79 (70.5) | 72 (82.8) |  |
| No specific interval recommended | 9 (9.7) | 26 (12.6) |  | 10 (9.9) | 16 (14.3) | 9 (10.3) |  |
| **Interval Between New Foods**  **(allergic)** |  |  |  |  |  |  |  |
| <3 d | 2 (2.2) | 7 (3.4) | 0.657 | 3 (3.0) | 5 (4.5) | 1 (1.1) | 0.292 |
| ≥3 d | 87 (93.5) | 187 (90.3) |  | 90 (89.1) | 104 (92.9) | 80 (92.0) |  |
| No specific interval recommended | 4 (4.3) | 13 (6.3) |  | 8 (7.9) | 3 (2.7) | 6 (6.9) |  |
| **CF Initiation Method** |  |  |  |  |  |  |  |
| Spoon-fed as purée | 64 (68.8) | 100 (48.3) | **0.004** | 43 (42.6) | 58 (51.8) | 63 (72.4) | **<0.001** |
| BLW ^‡^ | 1 (1.1) | 2 (1.0) |  | 1 (1.0) | 1 (0.9) | 1 (1.1) |  |
| BLW and spoon-fed puree | 16 (17.2) | 60 (29.0) |  | 35 (34.7) | 31 (27.7) | 10 (11.5) |  |
| Spoon-fed as textured | 12 (12.9) | 45 (21.7) |  | 22 (21.8) | 22 (19.6) | 13 (14.9) |  |
| **Recommendation of BLW** |  |  |  |  |  |  |  |
| No | 29 (31.2) | 41 (19.8) | **0.031** | 14 (13.9) | 20 (17.9) | 36 (41.4) | **<0.001** |
| Yes | 64 (68.8) | 166 (80.2) |  | 87 (86.1) | 92 (82.1) | 51 (58.6) |  |
| **Recommendation of BLISS** |  |  |  |  |  |  |  |
| No | 7 (7.5) | 12 (5.8) | 0.828 | 7 (6.9) | 5 (4.5) | 7 (8.0) | 0.663 |
| Yes | 34 (36.6) | 80 (38.6) |  | 41 (40.6) | 39 (34.8) | 34 (39.1) |  |
| Not familiar | 52 (55.9) | 115 (55.6) |  | 53 (52.5) | 68 (60.7) | 46 (52.9) |  |

mo: month; d: day; CF: complementary feeding; BLW: baby-led weaning; BLISS: Baby-Led Introduction to SolidS.

† The “Other” category was excluded from the analysis for “Total breastfeeding duration” due to insufficient sample size.
‡ Due to the limited number of respondents selecting only BLW, these cases were grouped with “BLW and spoon-fed purées” for analysis.

**Table S5.** Complementary Feeding Recommendations by Workplace Setting and Professional Title

|  | ***Workplace n (%)*** | |  | **Professional Title n (%)** | | |  |
| --- | --- | --- | --- | --- | --- | --- | --- |
|  | **Public** | **Private** | ***p* value** | **Pediatric**  **residents** | **General**  **pediatrician** | **Pediatric**  **subspecialist** | ***p* value** |
| **Exclusive Breastfeeding Duration** |  |  |  |  |  |  |  |
| 4-6 mo | 16 (8.3) | 13 (12.1) | 0.279 | 5 (13.9) | 20 (8.7) | 4 (11.4) | 0.492 |
| 6 mo | 177 (91.7) | 94 (87.9) |  | 31 (86.1) | 209 (91.3) | 31 (88.6) |  |
| **Total Breastfeeding Duration** |  |  |  |  |  |  |  |
| 12-24 mo | 11 (5.7) | 7 (6.5) | 0.280 | 4 (11.1) | 13 (5.7) | 1 (2.9) | 0.495 |
| 24 mo | 145 (75.1) | 87 (81.3) |  | 29 (80.6) | 175 (76.4) | 28 (80.0) |  |
| At least 24 | 27 (14.0) | 7 (6.5) |  | 2 (5.6) | 29 (12.7) | 3 (8.6) |  |
| >24 | 8 (4.1) | 5 (4.7) |  | 1 (2.8) | 9 (3.9) | 3 (8.6) |  |
| Other ^†^ | 2 (1.0) | 1 (0.9) |  | 0 (0.0) | 3 (1.3) | 0 (0.0) |  |
| **Timing of CF (non-allergic)** |  |  |  |  |  |  |  |
| <6 mo | 11 (5.7) | 12 (11.2) | 0.205 | 2 (5.6) | 17 (7.4) | 4 (11.5) | 0.893 |
| 6 mo | 172 (89.1) | 91 (85.0) |  | 32 (88.9) | 201 (87.8) | 30 (85.7) |  |
| >6 mo | 10 (5.2) | 4 (3.7) |  | 2 (5.6) | 11 (4.8) | 1 (2.9) |  |
| **Timing of CF (allergic)** |  |  |  |  |  |  |  |
| <6 mo | 24 (12.5) | 22 (20,6) | 0.093 | 5 (13.9) | 37 (16.1) | 4 (11.5) | 0.528 |
| 6 mo | 147 (76.2) | 78 (72,9) |  | 25 (69.4) | 173 (75.5) | 27 (77.1) |  |
| >6 mo | 22 (11.4) | 7 (6,5) |  | 6 (16.7) | 19 (8.3) | 4 (11.4) |  |
| **Interval Between New Foods**  **(non-allergic)** |  |  |  |  |  |  |  |
| <3 d | 20 (10.4) | 10 (9.3) | 0.831 | 3 (8.3) | 21 (9.2) | 6 (17.1) | 0.196 |
| ≥3 d | 152 (78.8) | 83 (77.6) |  | 32 (88.9) | 177 (77.3) | 26 (74.3) |  |
| No specific interval recommended | 21 (10.9) | 14 (13.1) |  | 1 (2.8) | 31 (13.5) | 3 (8.6) |  |
| **Interval Between New Foods**  **(allergic)** |  |  |  |  |  |  |  |
| <3 d | 6 (3.1) | 3 (2.8) | 0.845 | 1 (2.8) | 7 (3.1) | 1 (2,9) | 0.972 |
| ≥3 d | 175 (90.7) | 99 (925) |  | 34 (94.4) | 208 (90.8) | 32 (91,4) |  |
| No specific interval recommended | 12 (6.2) | 5 (4.7) |  | 1 (2.8) | 14 (6.1) | 2 (5,7) |  |
| **CF Initiation Method** |  |  |  |  |  |  |  |
| Spoon-fed as purée | 98 (50.8) | 66 (61.7) | 0.183 | 20 (55.6) | 124 (54.1) | 20 (57.1) | 0.989 |
| BLW ^‡^ | 3 (1.6) | 0 (0.0) |  | 0 (0.0) | 3 (1.3) | 0 (0.0) |  |
| BLW and spoon-fed puree | 53 (27.5) | 23 (21.5) |  | 10 (27.8) | 57 (24.9) | 9 (25.7) |  |
| Spoon-fed as textured | 39 (20.2) | 18 (16.8) |  | 6 (16.7) | 45 (19.7) | 6 (17.1) |  |
| **Recommendation of BLW** |  |  |  |  |  |  |  |
| No | 45 (23.3) | 25 (23.4) | 0.992 | 5 (13.9) | 52 (22.7) | 13 (37.1) | 0.062 |
| Yes | 148 (76.7) | 82 (76.6) |  | 31 (86.1) | 177 (77.3) | 22 (62.9) |  |
| **Recommendation of BLISS** |  |  |  |  |  |  |  |
| No | 14 (7.3) | 5 (4.7) | 0.676 | 0 (0.0) | 15 (6.6) | 4 (11.4) | **0.012** |
| Yes | 73 (37.8) | 41 (38.3) |  | 22 (61.1) | 83 (36.2) | 9 (25.7) |  |
| Not familiar | 106 (54.9) | 61 (57.0) |  | 14 (38.9) | 131 (57.2) | 22 (62.9) |  |

mo: month; d: day; CF: complementary feeding; BLW: baby-led weaning; BLISS: Baby-Led Introduction to SolidS.

† The “Other” category was excluded from the analysis for “Total breastfeeding duration” due to insufficient sample size.
‡ Due to the limited number of respondents selecting only BLW, these cases were grouped with “BLW and spoon-fed purées” for analysis.

**Table S6.** Logistic Regression Model Summaries

| **Model Outcome** | **Chi-square (df), p** | **-2 Log Likelihood** | **Nagelkerke R²** | **Hosmer-Lemeshow χ² (df), p** |
| --- | --- | --- | --- | --- |
| **BLW Recommendation** | 28.79 (6), p < 0.001 | 297.176 | 0.138 | 8.18 (7), p = 0.317 |
| **BLISS Recommendation** | 12.52 (6), p = 0.051 | 385.923 | 0.056 | 2.63 (8), p = 0.955 |
| **CF initiation with BLW and Spoon-fed puree** | 19.30 (6), p = 0.004 | 326.607 | 0.091 | 6.97 (7), p = 0.432 |
| **CF initiation with spoon-fed puree only** | 30.25 (6), p < 0.001 | 383.024 | 0.128 | 8.34 (7), p = 0.303 |
