# Supplementary material for: Clinical variability in complementary feeding counseling in Türkiye: results from a pediatrician survey
Source: Front Pediatr. 2025 Nov 11;13:1646667. doi: 10.3389/fped.2025.1646667 (PMC12644083; doi:10.3389/fped.2025.1646667)
Supplement: Supplementary file 2 [file Supplementaryfile2.docx]

**Complementary Feeding Recommendations and Practices Questionnaire**

Dear Colleague,

Early-life nutrition plays a critical role in shaping long-term metabolic health and body composition. Complementary feeding is therefore essential to ensure a healthy start in life. Despite the existence of international guidelines, parents often rely on informal sources such as blogs, books, or family advice. Moreover, healthcare professionals may also have diverse approaches. This study aims to investigate pediatricians’ recommendations and practices regarding the introduction of complementary foods.

Participation is voluntary, and all responses will be anonymized and used solely for academic purposes.

Do you consent to participate in the study?

□ Yes □ No

**SECTION 1 –Demographic and Professional Information**

1. Age

_____

1. Gender
   □ Male □ Female
2. Professional Title
   □ Pediatric Resident

□ General Pediatrician

□ Pediatric Subspecialist

1. Years of experience

_____ years

1. Subspecialty

□ Emergency & Intensive Care

□ Allergy

□ Endocrinology

□ Gastroenterology

□ Developmental Pediatrics

□ Genetics

□ Pulmonology

□ Hematology/Oncology

□ Infectious Diseases

□ Cardiology

□ Metabolism

□ Nephrology

□ Neurology

□ Rheumatology

□ Social Pediatrics

□ Neonatology

1. Type of institution
   □ State Hospital

□ Research and Training Hospital

□ City Hospital

□ University Hospital

□ Private Hospital

□ Private Practice

1. Province of practice

_____

**SECTION 2 – Breastfeeding and Introduction of Complementary Foods**

1. For how many months do you recommend exclusive breastfeeding?

_____ months

1. For how many months do you recommend total breastfeeding (exclusive and continued)?

_____ months

1. Which food group do you recommend introducing first during complementary feeding?

□ Vegetables

□ Fruit juice

□ Fruit purée

□ Yogurt

□ Cereal

□ Egg yolk

□ Cheese

□ Puréed red meat

□ Pudding

1. At what age do you recommend introducing vegetables?

□ <4 mo

□ 4 mo

□ 5 mo

□ 6 mo

□ 7 mo

□ 7–9 mo

□ 9–12 mo

□ >12 mo

1. Which vegetables do you recommend introducing first? *(Select all that apply)*

□ Potato

□ Sweet potato

□ Carrot

□ Zucchini

□ Pumpkin

□ Broccoli

□ Cauliflower

□ Celery

□ Tomato

□ Pepper

□ Beetroot

1. At what age do you recommend introducing fruits?

□ <4 mo

□ 4 mo

□ 5 mo

□ 6 mo

□ 7 mo

□ 7–9 mo

□ 9–12 mo

□ >12 mo

1. Which fruits do you recommend introducing first? *(Select all that apply)*

□ Banana

□ Apple

□ Peach

□ Orange

□ Mandarin

□ Pear

□ Grape

□ Plum

□ Watermelon

□ Melon

□ Avocado

□ Kiwi

□ Mango

□ Other tropical fruits

1. Do you recommend yogurt during complementary feeding?

□ Yes □ No

1. If yes, at what age do you recommend introducing yogurt?

□ <4 mo

□ 4 mo

□ 5 mo

□ 6 mo

□ 7 mo

□ 7–9 mo

□ 9–12 mo

□ >12 mo

1. Do you recommend red meat during complementary feeding?

□ Yes □ No

1. If yes, at what age do you recommend introducing red meat?

□ <4 mo

□ 4 mo

□ 5 mo

□ 6 mo

□ 7 mo

□ 7–9 mo

□ 9–12 mo

□ >12 mo

1. Do you recommend chicken during complementary feeding?

□ Yes □ No

1. If yes, at what age do you recommend introducing chicken?

□ <4 mo

□ 4 mo

□ 5 mo

□ 6 mo

□ 7 mo

□ 7–9 mo

□ 9–12 mo

□ >12 mo

1. At what age do you recommend introducing cheese?

□ <4 mo

□ 4 mo

□ 5 mo

□ 6 mo

□ 7 mo

□ 7–9 mo

□ 9–12 mo

□ >12 mo

1. At what age do you recommend introducing olive?

□ <4 mo

□ 4 mo

□ 5 mo

□ 6 mo

□ 7 mo

□ 7–9 mo

□ 9–12 mo

□ >12 mo

1. At what age do you recommend introducing molasses?

□ <4 mo

□ 4 mo

□ 5 mo

□ 6 mo

□ 7 mo

□ 7–9 mo

□ 9–12 mo

□ >12 mo

1. At what age do you recommend introducing honey?

□ <4 mo

□ 4 mo

□ 5 mo

□ 6 mo

□ 7 mo

□ 7–9 mo

□ 9–12 mo

□ >12 mo

1. At what age do you recommend introducing legumes *(e.g., lentils, beans, chickpeas)?*

□ <4 mo

□ 4 mo

□ 5 mo

□ 6 mo

□ 7 mo

□ 7–9 mo

□ 9–12 mo

□ >12 mo

1. Do you recommend introducing nuts during complementary feeding?

□ Yes □ No

1. If yes, at what age do you recommend introducing nuts?

□ <4 mo

□ 4 mo

□ 5 mo

□ 6 mo

□ 7 mo

□ 7–9 mo

□ 9–12 mo

□ >12 mo

1. Do you recommend foods that contain refined sugar (e.g., table sugar, packaged sweets)?

□ Yes □ No

1. If yes, from which month?

□ <4 mo

□ 4 mo

□ 5 mo

□ 6 mo

□ 7 mo

□ 7–9 mo

□ 9–12 mo

□ >12 mo

1. At what age do you recommend introducing salt?

□ <4 mo

□ 4 mo

□ 5 mo

□ 6 mo

□ 7 mo

□ 7–9 mo

□ 9–12 mo

□ >12 mo

1. At what age do you recommend introducing water?

□ <4 mo

□ 4 mo

□ 5 mo

□ 6 mo

□ 7 mo

□ 7–9 mo

□ 9–12 mo

□ >12 mo

1. Do you recommend introducing plant-based milk during complementary feeding?

□ Yes □ No

1. If yes, from which month?

□ <4 mo

□ 4 mo

□ 5 mo

□ 6 mo

□ 7 mo

□ 7–9 mo

□ 9–12 mo

□ >12 mo

1. Do you recommend introducing herbal teas (e.g., linden) during complementary feeding?

□ Yes □ No

1. If yes, at what age do you recommend introducing herbal teas?

□ <4 mo

□ 4 mo

□ 5 mo

□ 6 mo

□ 7 mo

□ 7–9 mo

□ 9–12 mo

□ >12 mo

1. Do you think children can drink black tea?

□ Yes □ No

1. If yes, from which month?

□ <4 mo

□ 4 mo

□ 5 mo

□ 6 mo

□ 7 mo

□ 7–9 mo

□ 9–12 mo

□ >12 mo

1. Do you think children can drink coffee?

□ Yes □ No

1. If yes, from what age?

□ 1 yr

□ 2 yr

□ 3 yr

□ 4 yr

□ 4–6 yr

□ >6 yr

1. Do you think children can drink mineral water?

□ Yes □ No

1. If yes, from what age?

□ 6–12 mo

□ 1 yr

□ 2 yr

□ 3 yr

□ 3–6 yr

□ 6–12 yr

□ >12 yr

**SECTION 3 –Complementary Feeding Recommendations in Allergic and Non-Allergic Infants**

*In this section “Allergic infants” refers to infants with any type of physician-diagnosed allergy condition (e.g. atopic dermatitis) except specific food allergies.“Non- allergic infants” refers to infants without any history of allergic conditions.*

1. When do you recommend starting complementary feeding in non-allergic infants?

□ <4 mo

□ 4 mo

□ 5 mo

□ 6 mo

□ 6–9 mo

□ >9 mo

1. When do you recommend starting complementary feeding in allergic infants?

□ <4 mo

□ 4 mo

□ 5 mo

□ 6 mo

□ 6–9 mo

□ >9 mo

1. How many days apart do you recommend introducing new foods to non-allergic infants?

□ 1 day

□ 2 days

□ 3–5 days

□ 5–7 days

□ No specific interval needed

1. How many days apart do you recommend introducing new foods to allergic infants?

□ 1 day

□ 2 days

□ 3–5 days

□ 5–7 days

□ No specific interval needed

1. When do you recommend introducing egg white in non-allergic infants?

□ <4 mo

□ 4 mo

□ 5 mo

□ 6 mo

□ 7 mo

□ 7–9 mo

□ 9–12 mo

□ >12 mo

1. When do you recommend introducing egg white in allergic infants?

□ <4 mo

□ 4 mo

□ 5 mo

□ 6 mo

□ 7 mo

□ 7–9 mo

□ 9–12 mo

□ >12 mo

1. When do you recommend introducing egg yolk in non-allergic infants?

□ <4 mo

□ 4 mo

□ 5 mo

□ 6 mo

□ 7 mo

□ 7–9 mo

□ 9–12 mo

□ >12 mo

1. When do you recommend introducing egg yolk in allergic infants?

□ <4 mo

□ 4 mo

□ 5 mo

□ 6 mo

□ 7 mo

□ 7–9 mo

□ 9–12 mo

□ >12 mo

1. Do you recommend cow’s milk during complementary feeding?

□ Yes □ No

1. When do you recommend introducing cow’s milk in non-allergic infants?

□ <4 mo

□ 4 mo

□ 5 mo

□ 6 mo

□ 7 mo

□ 7–9 mo

□ 9–12 mo

□ 12–24 mo

□ >24 mo

1. When do you recommend introducing cow’s milk in allergic infants?

□ <4 mo

□ 4 mo

□ 5 mo

□ 6 mo

□ 7 mo

□ 7–9 mo

□ 9–12 mo

□ 12–24 mo

□ >24 mo

1. Do you recommend fish during complementary feeding?

□ Yes □ No

1. When do you recommend introducing fish in non-allergic infants?

□ <4 mo

□ 4 mo

□ 5 mo

□ 6 mo

□ 7 mo

□ 7–9 mo

□ 9–12 mo

□ >12 mo

1. When do you recommend introducing fish in allergic infants?

□ <4 mo

□ 4 mo

□ 5 mo

□ 6 mo

□ 7 mo

□ 7–9 mo

□ 9–12 mo

□ >12 mo

1. Do you recommend cereals during complementary feeding?

□ Yes □ No

1. When do you recommend introducing cereals in non-allergic infants?

□ <4 mo

□ 4 mo

□ 5 mo

□ 6 mo

□ 7 mo

□ 7–9 mo

□ 9–12 mo

□ >12 mo

1. When do you recommend introducing cereals in allergic infants?

□ 4 mo

□ 5 mo

□ 6 mo

□ 7 mo

□ 7–9 mo

□ 9–12 mo

□ >12 mo

1. When do you recommend introducing peanut in allergic infants?

□ <4 mo

□ 4 mo

□ 5 mo

□ 6 mo

□ 7 mo

□ 7–9 mo

□ 9–12 mo

□ >12 mo

**SECTION 4 -Feeding Methods and Textural Progression**

1. What method do you recommend for initiating complementary feeding?

□ Spoon-fed purée

□ Spoon-fed mashed/lumpy

□ BLW (Baby-Led Weaning) method

□ Combination of BLW and spoon feeding

1. At what age do you recommend introducing lumpy foods?

□ 6 mo

□ 6–9 mo

□ 9–12 mo

□ >12 mo

1. At what age do you recommend introducing finger foods?

□ 6 mo

□ 6–9 mo

□ 9–12 mo

□ >12 mo

1. At what age do you recommend transitioning to family meals?

□ 6 mo

□ 6–9 mo

□ 9–12 mo

□ >12 mo

1. Do you recommend commercially prepared jarred baby foods?

□ Yes □ No

1. Do you recommend the Baby-Led Weaning (BLW) method?

□ Yes □ No

1. If yes, at what age do you recommend starting BLW?

□ 6 mo

□ 6–9 mo

□ 9–12 mo

□ >12 mo

1. Do you recommend the Baby-Led Introduction to Solids (BLISS) method?

□ Yes □ No

□ Not familiar with this method

1. If yes, at what age do you recommend starting BLISS?

□ 6 mo

□ 6–9 mo

□ 9–12 mo

□ >12 mo
